# Identification of selected genes associated with the SARS-CoV-2: a therapeutic approach and disease severity

**DOI:** 10.1186/s42269-021-00540-y

**Published:** 2021-04-23

**Authors:** Ramakrishnan Veerabathiran, Barath Ragunath, Vaishak Kaviarasan, Vajagathali Mohammed, Shiek S. S. J. Ahmed

**Affiliations:** 1 Human Cytogenetics and Genomics Laboratory, Faculty of Allied Health Sciences, Chettinad Hospital and Research Institute (CHRI) Chettinad Academy of Research and Education (CARE), Kelambakkam, Tamilnadu 603103 India; 2 Drug Discovery and Multi-Omics Laboratory, Faculty of Allied Health Sciences, Chettinad Hospital and Research Institute (CHRI) Chettinad Academy of Research and Education (CARE), Kelambakkam, Tamilnadu 603103 India

**Keywords:** SARS-CoV-2, Genes, ACE2, Interleukin, Cytokines

## Abstract

**Background:**

The ongoing pandemic of COVID-19 viruses takes its sole origin from the Wuhan Huanan seafood market, China. The first case was recorded as viral pneumonia and later became a worldwide pandemic (officially declared by WHO on March 11, 2020).

**Main body:**

SARS-CoV-2 is an extremely infectious and transferrable virus that develops severe conditions like respiratory syndrome, high blood pressure and weakens the immune system. Coronavirus falls under the Coronaviridae family and Beta coronavirus genus. Affected individuals will encounter problems starting with fever followed by severe complications like SARS, ARDS, and many others. These SARS-CoV and MERS-CoV enter the host cells by the endosomal pathway, and about 16 non-structural proteins are involved in assembling the viral RNA synthesis complex. They possess a positive-sense single-stranded RNA, and about four major genes are mainly associated with the development of ASRD, SARS, and other respiratory problems.

**Conclusion:**

Susceptibility of these four major genes such as ACE2, IL-2, 7 and 10, TNF, and VEGF is associated with COVID-19. This highlights the identification of the above-mentioned genes that can be used as potential biomarkers for early diagnosis and targeted drug delivery for treating the SARS-CoV-2 neurological symptoms and reducing inflammation in the brain.

## Background

**Fig. 1:**
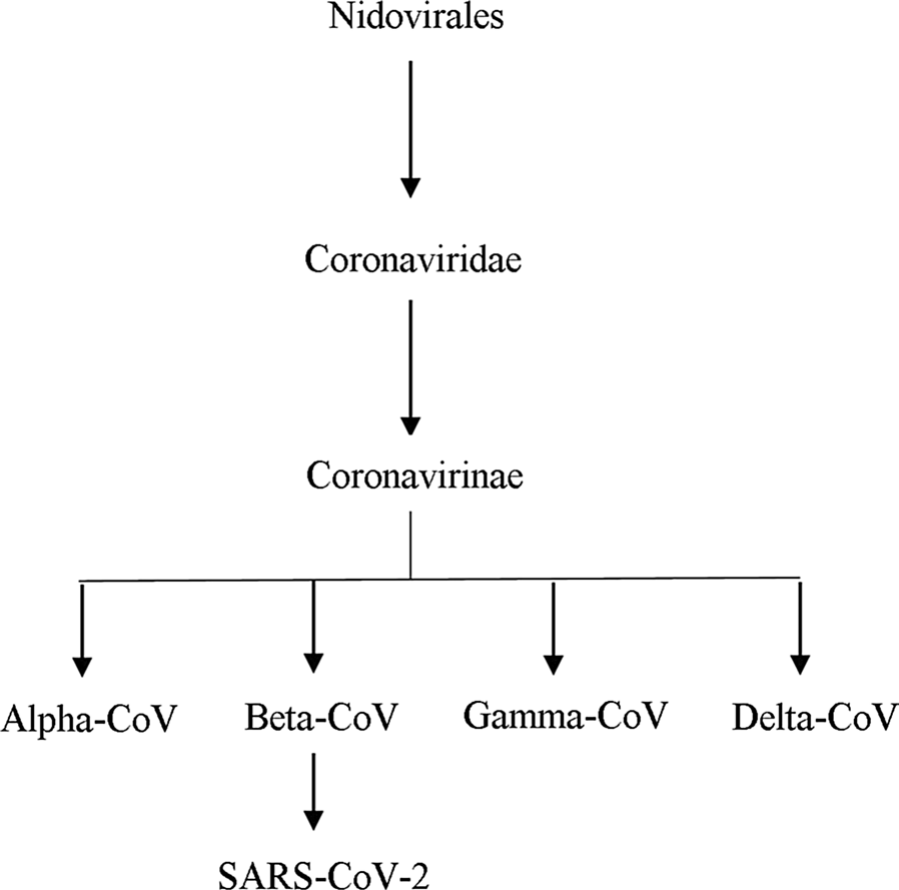
Hierarchy of Nidovirales

Coronavirus was first likely to emerge in Wuhan city, Hubei Province, China. It is suspected that its transmission was from the animal host and then spread to humans (Zhu et al. 2020). SARS-CoV-2 matches 79% to SARS-CoV and 50% to MERS-CoV, it even has a high genetic similarity with bat CoV (RaTG13), but bats are not a primary source coronavirus. Common symptoms of SARS-CoV-2 are fever, cough, and sore throat (Poon et al. 2005). This virus is highly contagious and can transmit faster. According to the laws of IHR, 2005, declared a PHEIC because the spread was among 18 countries on January 30, 2020 (Cascella et al. 2020). CoV belongs to *Nidovirales* order, a family of *Coronaviridae* and *Orthocoronavirinae* subfamily; it is divided further into four groups according to genera are as follows: alpha (*α*), beta (*β*), gamma (*γ*), delta (*δ*) (Fig. 1) (Fehr and Perlman 2015). The purpose of this article is to explain the prevalence, risk factors, life cycle, structure, genetic aspects, and significant genes involved and associated with SARS-CoV-2.

## Prevalence

The ongoing pandemic COVID-19 started to emerge in December 2019. The severity and adverse effects increased gradually, so WHO declared it as a pandemic (Worldwide) on March 11, 2020 (Al-Tawfiq et al. 2020). The more familiar manifestation among the affected individuals is fever followed by several associated complications like diarrhea and severe body pain, which leads to the cruelty of the infection (Hu et al. 2020). Beginning (March 3, 2020), 73 countries, territories, or areas worldwide had been encountered this infection and affected about 90,870 individuals. Studies say that these pandemics may be related to bats, but it takes their sole origin from the Wuhan Huanan seafood market (Ge et al. 2020).

**Fig. 2:**
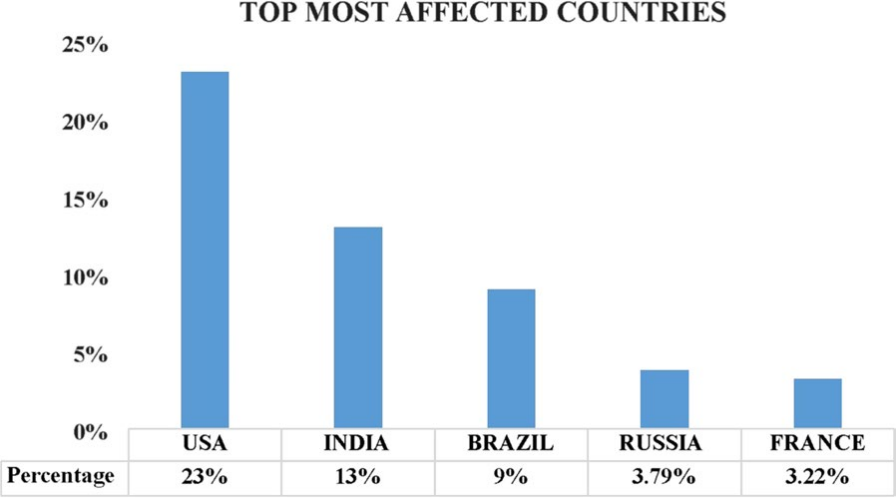
Graphical representation of topmost affected countries worldwide till December 20, 2020 (WHO dashboard report covid19.who.int)

According to WHO, as of December 20, 2020 (3.05 pm), globally the total number of COVID confirmed cases rose to 75,098,369, and the total no deaths across the globe reaches 1,680,339. Figure 2 shows the USA stands first in position among the COVID confirmed cases across the world and contributes to 23.05% of all confirmed cases with a mortality rate of 18.51% followed by India with 13.35% and 8.65%, Brazil—9.53% and 11.04%, Russia—3.79% and 3.02%, France—3.22% and 3.57%. Some of the least affected countries are Denmark with 0.17% confirmed cases and mortality rate of about 0.06%, Malaysia—0.12% and 0.025%, Norway—0.056% and 0.024%, Finland—0.043% and 0.029% and Zimbabwe with 0.016% and 0.018%, etc.

## Risk factors

**Fig. 3:**
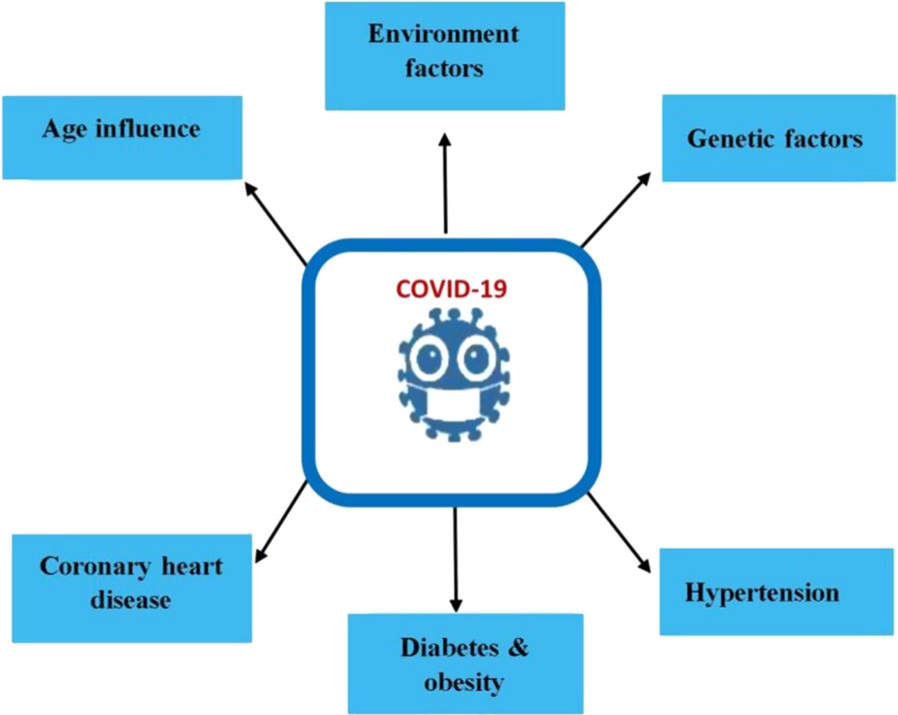
Multiple risk factors of COVID-19

Figure 3 demonstrated the risk factors that can cause death in COVID-19 are older having hypertension and diabetes mellitus, and the majority of those affected were males compared to females (Wolff 2020). The severity of this disease may progress due to various lifestyle factors such as smoking, obesity, staying for a longer time in a hospital for admission may expose us to disease, and being unhygienic (Chou et al. 2020; Rod et al. 2020).

There are eight comorbidities evolved during SARS-CoV-2 infection on disease severity; they are as follows: tissue failure, acute respiratory distress syndrome (ARDS), severe pneumonia, immunological dysfunction, uncontrolled inflammation response, acute liver injury, hypoproteinemia, and hypercoagulable state (Zaim et al. 2020).

## Structure of coronavirus

Coronavirus was named because of its halo (coronas) structure when seen under an electron microscope, and these belong to the RNA virus family (Chan et al. 2013). These are non-segmented, positive-sense RNA with a genome of size ~ 30 kb and allowing it to play as mRNA for translation of the replicase polyproteins because of cap structure in 5′ and poly-A tail in 3` ends (Perlman and Netland 2009).

**Fig. 4:**
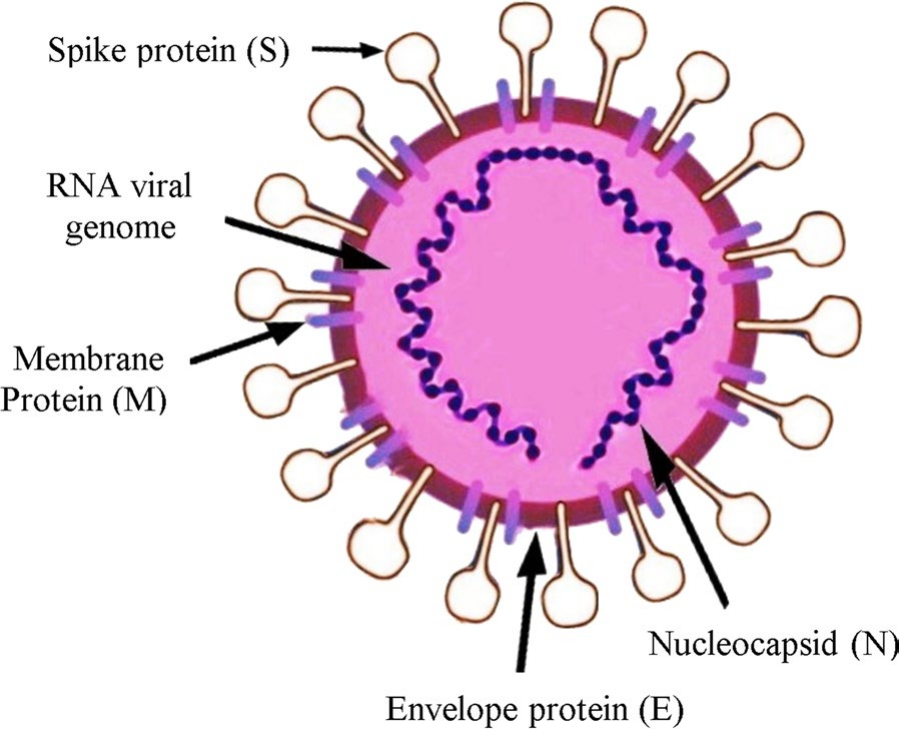
Structure of human SARS-CoV-2 virus

Coronavirus (COVID-19) is consisting of four main viral structural proteins that consist of Spike, Membrane, Envelope, and Nucleocapsid shown in Fig. 4.

## Spike glycoprotein

Coronavirus S proteins have a large group of multi-functional viral transmembrane proteins of class I that have 1160–1400 amino acids (Li et al. 2019). The crucial immune-dominant proteins of CoV’s are S proteins which can induce the host’s immune responses (Li et al. 2003).

## Membrane protein

The protein is present in ample amounts inside the virion particles, and it gives a proper shape to the viral envelope (Ziebuhr 2005).

## Envelope protein

The smallest among the main structural proteins is the Envelope protein (Brian and Baric 2005). This protein also has a crucial role in pathogenesis, connecting, and viral departure (Fischer et al. 1998).

## Nucleocapsid protein

This protein also has multi-functions, among other functions that play a vital role in complex formation with viral genome and enhances assembly in M protein (Zúñiga et al. 2007; Frieman and Baric 2008).

## Life cycle and process of SARS-CoV-2

**Fig. 5:**
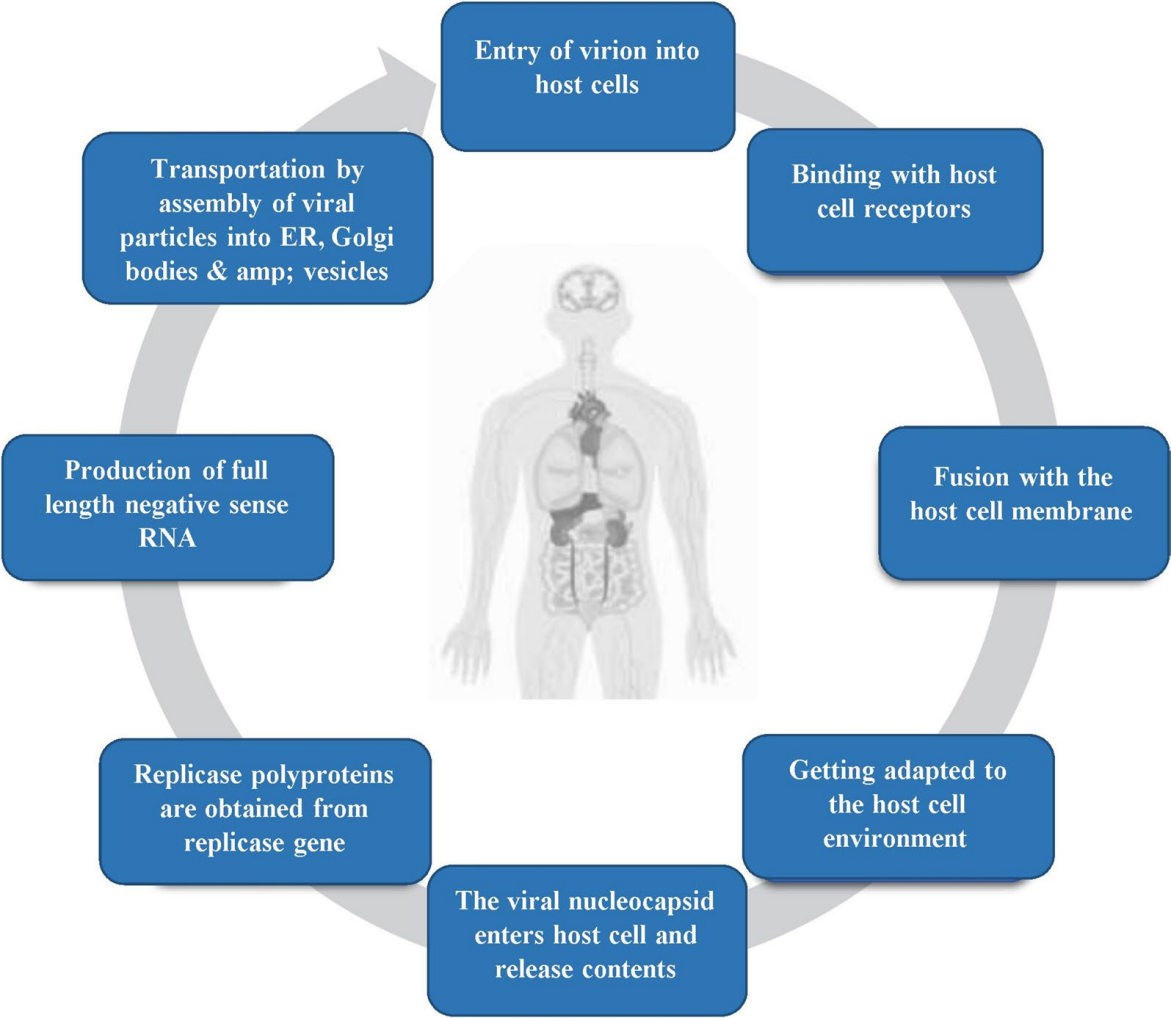
Life cycle of SARS-CoV-2

The life cycle of the novel coronavirus begins with the arrival of the virion into the cell that is being invaded. The cell entry is facilitated by glycoprotein spikes present in the structure SARS-COVID and binds with the receptors of the host cell, and the process is called host cell recognition. The ability to withstand the new host cell environment and to escape from the human immune system is also provided by the spikes (Kirchdoerfer et al. 2018; Perlman and Netland 2009). The cellular proteases like Cathepsins, the human airway trypsin-like protease (HAT), TMPRSS2 facilitates the entry of these virions by separating the spike proteins and further building up additional perforation changes (Glowacka et al. 2011; Bertram et al. 2011) (Fig. 5).

The SARS-COVID requires ACE2 as a critical receptor for cell entry, whereas MERS-COVID requires dipeptidyl peptidase (DPP4) (Wang et al. 2013; Raj et al. 2013). After entering the cell, the RNA gets uncoated, and two replicase polyproteins are obtained by translation of the replicase gene present on the RNA strand. Also, the unique proteins of replicase enzymes are obtained by further processing the proteinases of the virus. Moreover, these proteins result in full-length RNA of negative sense, which is further reproduced to distribute shorter mRNAs (Song et al. 2019). Then, the genomic RNA and the viral proteins are further assembled into virions in the Golgi bodies and endoplasmic reticulum, and the vesicles help in the transportation of these particles and are released outside the cell. (Shereen et al. 2020).

## Genetic aspects of COVID-19

COVID-19 possesses an ssRNA (positive sense) linked with a nucleoprotein inside a capsid consisting of matrix protein. It consists of the largest genome (26.4–31.7 kb) than other viruses known till now (Mousavizadeh and Ghasemi 2020). Previous studies propose that ARDS is more likely to be developed in all instances, such as in MERS-CoV, SARS-CoV, and SARS-CoV-2 (Ding et al. 2003). In numerous patients, the genes like TNF, ACE2, IL-10, and VEGF are considered to be involved with the progress of ARDS (Meyer and Christie 2013). Even though this SARS-CoV-2 belongs to the SARS family, it slightly differs from SARS-CoV in Envelope (E), Membrane (M), Nucleocapsid, and Spike proteins which are given in Table 1 (Rehman et al. 2020).

**Table 1:** Protein variations and similarities between SARS-CoV and SARS-CoV-2 (Rehman et al. 2020)

Proteins	Similarities (%)	Genetic variations (%)
Envelope protein	93	7
Membrane protein	92	8
Nucleocapsid protein	93	7
Spike protein	81	19

Genetic studies revealed that DNA polymorphisms in TMPRSS2/ACE2 were more likely correlated with a genetic vulnerability to SARS-CoV2, so interpreting these studies will be more valuable for developing vaccines (Hou et al. 2020). Gene interaction with environmental factors like smoking and lifestyle activities is considered. Also, there is a high susceptibility of COVID-19. In COVID-19 infection, SARS-CoV is suspected of playing a significant role in genetic predisposition because it matches 80 percent of the genetic identity (Darbeheshti and Rezaei 2020).

## Methodology

The genes mentioned in this study are identified from past 25-year literature papers from Web of Science, PubMed, and several other databases. All the literature was selected based on the title and abstract, and two independent authors referred to the published papers according to the content. The articles were separated into three groups. The first, second, and third group articles were collected according to the SARS-CoV-2 context, followed by gene, and genetic polymorphism articles were addressed gene expressions and their association with the disease. The process of collecting the relevant articles for this review is shown in Fig. 2. The selected genes have a mutation in both intronic and exonic regions and identified genes' expression in various locations. The following is a list of selected genes which is associated with the SARS-CoV-2.

## Major genes associated with COVID-19

There are various SNPs and genes associated with COVID-19. In this review, we empathize with the current scenario, recent advancements, and enduring challenges of the susceptibility of four significant genes [ACE2, (IL-2, 7, 10), TNF, and VEGF] associated with COVID-19 are progressively involved in the development of ASRD, SARS, and other respiratory problems according to their function (Table 2).

**Table 2:** Significant genes involved in COVID-19

S. no.	Genes	Chromosome location	Total exons	Function	Clinical significance	References
1.	Angiotensin-converting enzyme-2 (ACE-2)	Xp22.2	20	Functional receptor for the spike glycoprotein of HCV-NL63, SARS, and SARS-CoV-2	It is a causative (COVID-19). It is under review for the study of coronavirus biology and its involvement of ACE-2 with SARS-CoV-2	www.ncbi.nlm.nih.gov
2. (a)	Interleukin-2 (IL-2)	4q27	4	Cytokine produced by activated CD4 + and CD8 + T lymphocytes that is important for the proliferation of T and B lymphocytes	IL-2 is under review for the study of coronavirus biology, and it is involved in cytokine storm inflammatory response	www.ncbi.nlm.nih.gov
(b)	Interleukin-7 (IL-7)	8q21.13	8	In association with the disease severity of COVID-19, circulating cytokines and chemokines have been found	IL-7 is under review for the study of coronavirus biology, and it is involved in cytokine storm inflammatory response	www.ncbi.nlm.nih.gov
(c)	Interleukin-10 (IL-10)	1q32.1	7	This cytokine can block NF-kappa B activity, and it is involved in the regulation of the JAK-STAT signaling pathway	IL-10 is under review for the study of coronavirus biology, and it is involved in cytokine storm inflammatory response	www.ncbi.nlm.nih.gov
3.	Tumor necrosis factor (TNF)	6p21.33	4	This cytokine regulates a broad spectrum of biological processes, including cell proliferation, differentiation, apoptosis, lipid metabolism, and coagulation	TNF is under review for the study of coronavirus biology, and it is involved in cytokine storm inflammatory response	www.ncbi.nlm.nih.gov
4.	Vascular endothelial growth factor (VEGF)	6p21.1	9	This growth factor induces proliferation and migration of vascular endothelial cells and is essential for both physiological and pathological angiogenesis	VEGF levels are higher during SARS-CoV-2 infection. This gene is under review for the study of coronavirus biology, and it is involved in cytokine storm inflammatory response	www.ncbi.nlm.nih.gov

## Interleukin (IL-2, 7, 10) Gene

IL-2 encodes a protein that secretes cytokine produced by activated CD4 + and CD8 + T lymphocytes essential for the T and B lymphocytes proliferation (www.ncbi.nlm.nih.gov). For B cells and T cells, and cytotoxic cells, IL-2 is a central mediator for growth and development, including natural killer and lymphokine-activated killer cells (Kasprzak and Olejniczak 2008). For proinflammatory cytokines and chemokines, SARS-CoV-2 infection can be a potent inducer of IL-7. In association with the severity of disease for COVID-19, circulating cytokines and chemokines have been found (www.ncbi.nlm.nih.gov). IL-7 plays a hectic role in the immune system's homeostasis, and it helps increase the healthspan by altering the immune system (Nguyen et al. 2017). This gene encodes a cytokine protein produced by monocytes primarily and a very few extents by lymphocytes and has a pleiotropic effect in immune-regulation and inflammation (www.ncbi.nlm.nih.gov). One of the required anti-inflammatory cytokines is IL-10; it plays a crucial role as a negative regulator to immune responses for microbial antigens. During the response to proinflammatory signals, immune cells can produce IL-10, and it also functions when there is excessive inflammation when infected (Iyer and Cheng xxxx). In severely sick COVID-19 patients, cytokine storm syndrome was examined, and it was reported that the extent of interleukins (IL-2, IL-7, IL-10) was high in fundamentally sick patients (Zwirner and Domaica 2010).

**Fig. 6:**
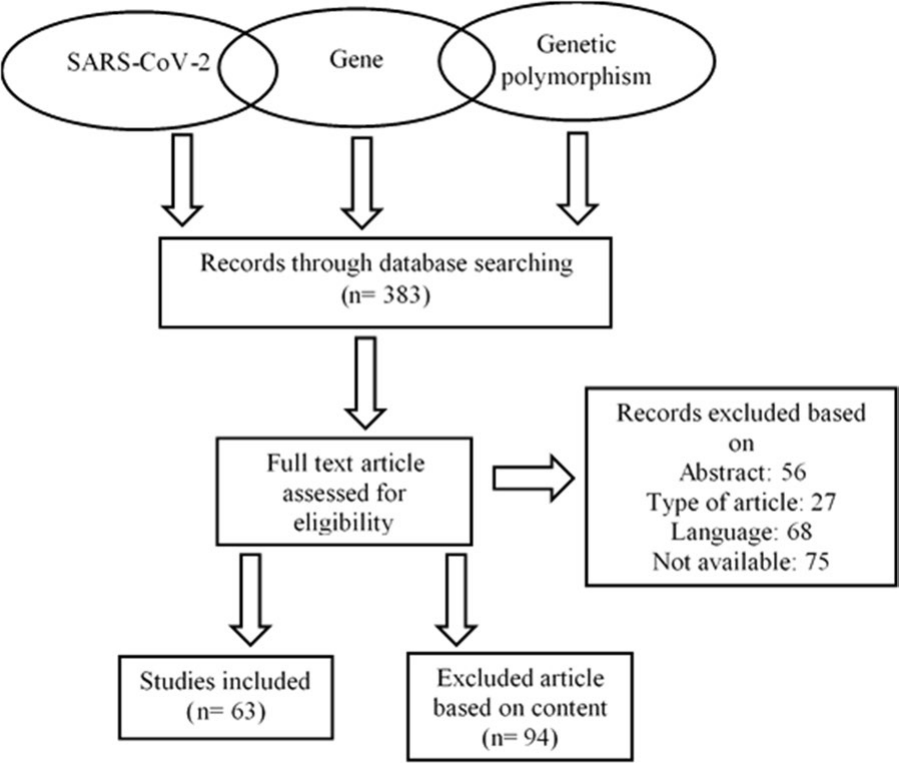
Study flow diagram

The proliferation of T, B, and NK cells in IL2 cytokine prevents autoimmune diseases (rheumatoid arthritis (RA), type 1 diabetes, multiple sclerosis) and does not initiate an autoimmune response (cell tolerance), shown in Fig. 6. In COVID-19 patients, the cytokine IL2 or its receptors (IL2R) elevated and increased the condition's severity (Costela-Ruiz et al. 2020).

**Fig. 7:**
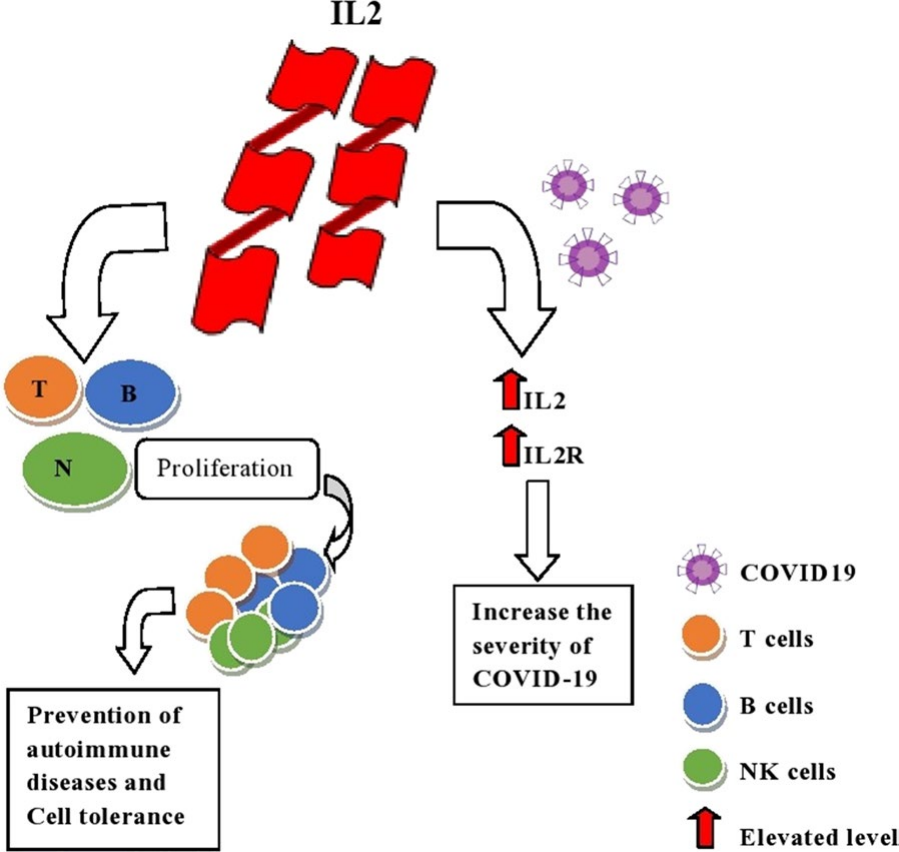
The severity of SARS-CoV-2 due to the interleukin-2 cytokine

IL7 plays a significant role in the WBC (lymphocytes) differentiation, and it activates the T cells, which regulates the negative transforming growth factor-beta (TGF β) in COVID-19 patient’s shown in Fig. 7. TGF-β transformation results to cause the elevation of IL7, and it directly increases the severity (Costela-Ruiz et al. 2020).

**Fig. 8:**
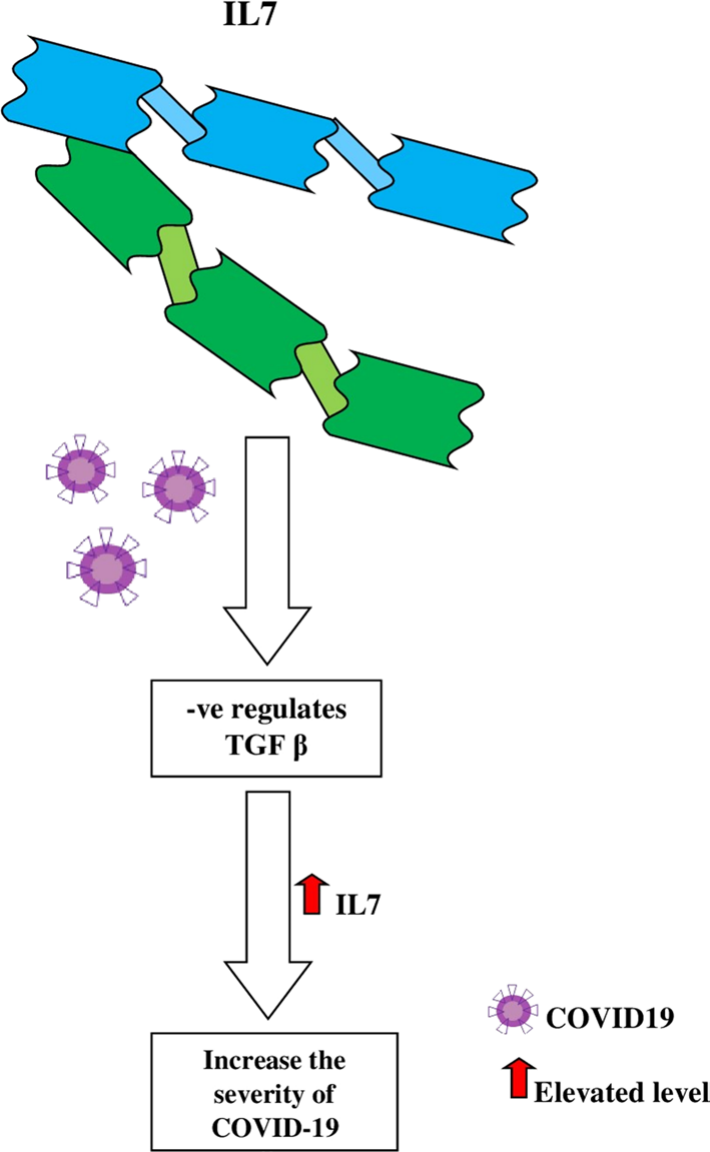
The severity of SARS-CoV-2 due to the interleukin-7 cytokine

In COVID-19 patients, the viral resistant is eliminated by inhibition of IL10, and it also blocks the IL10 signals shown in Fig. 8. It was found that the elderly patients were highly affected in COVID-19 by the hyperinflammatory causing the reduction of T cell receptors (Costela-Ruiz et al. 2020).

## ACE2 (angiotensin-converting enzyme-2) gene

This gene's encoded protein has a place with the angiotensin-changing over compound groups of dipeptidyl carboxypeptidases. It has a unique identity to human angiotensin one changing over chemical protein act as an active spike protein of SARS-CoV and SARS-CoV-2, the objective agent of COVID-19. The emitted protein catalyzes the cleavage of angiotensin one into angiotensin 1–9, and angiotensin II is converted into the vasodilator angiotensin 1–7. ACE2 is known to be communicated in various organs of the human body (www.ncbi.nlm.nih.gov). It has been accounted that ACE2 is the fundamental host cell receptor of 2019-nCoV and assumes a vital job in the passage of infection into the cell to cause the last contamination. Single-cell transcriptomes from free information created in-house were utilized to distinguish and affirm the ACE2-communicating cell synthesis and extent in the oral cavity (Wu et al. 2020). The outcomes exhibited that the ACE2 communicated on the mucosa of the oral cavity. For SARS-CoV-2, the essential receptor is ACE2 (in vivo); contaminations and the S proteins of the SARS-CoV-2 virus diminish ACE2 articulation (Xu et al. 2020).

## TNF (tumor necrosis factor) gene

These genes are the component of the TNF ligand superfamily that encodes for a proinflammatory cytokine, of which macrophages are mainly associated with these cytokine secretions and their specified locus on human chromosome 6p21.3 (El-Tahan et al. 2016). They get bound with similar TNF receptors and result in similar pleiotropic effects; many pathological processes are correlated with these types of the gene, including cell proliferation, cell death, immune regulation, and inflammatory responses (Boraska et al. 2010). The levels of TNF-α were found similarly higher in aged or older patients than others, and exhaustion in T cell checks is seen, which demonstrates that this TNF-α is a sort of negative regulator for the proliferation of T cells (Diao et al. 2019a). The plasma proportions of this TNF are found higher in SARS-COV-2-infected patients, and their concentrations are based on the severity of infection, which means higher concentrations are seen in ICU patients than in non-ICU patients (Diao et al. 2019a). The primary synthesizers of TNF called the macrophages are seen to a greater extent in the infected individuals; thus, these elevated levels of proinflammatory cytokines called the TNF are observed; in some patients, it turns out to be a cytokine storm (Soy et al. 2020). So different TNF blocking antibodies like etanercept, adalimumab, etc., are effectively employed to treat inflammatory diseases, and now these treatments are suggested for earnest requirements toward the COVID patients (Channappanavar et al. 2016).

## VEGF (vascular endothelial growth factor) gene

Eight conserved cysteines distinguish these genes; they also represent homodimer structures and functions. These are the protein types found with vascular permeability actions and are further subdivided into VEGF-A, B, C, D, E, PlGF, and Trimeresurus flavoviridis svVEGF (Shibuya 2011). This VEGF performs a prime part in maintaining the growth, improvement, and maintenance of a healthy circulatory system, thereby ensuring normal angiogenesis (Ruggiero et al. 2011). They get bound with VEGFR and perform a top part in the activating endothelial cell. The alveolar immune regulation is maintained by the integrity of the endothelial barrier in lung tissue which is crucial in COVID-affected patients (Zhang et al. 2020). In the SARS-CoV-2-affected individuals, the serum levels of VEGF are found to be elevated. However, there is not much difference in these VEGF levels between ICU and non-ICU patients (Huang et al. 2020). VEGF is mainly associated with ALI and ARDS and is believed to be a prime factor for their cause; since the proportions of these genes are found elevated in the COVID-19-infected persons, they may lead to acute lung and respiratory syndromes in affected individuals (Turkia). These VEGFs perform a decisive part in brain inflammation (which results in neurological defects) and is identified as a promising therapeutic agent in suppressing the inflammation that is caused by COVID infection (Yin et al. 2020; Rodríguez-Puertas 2020).

**Fig. 9:**
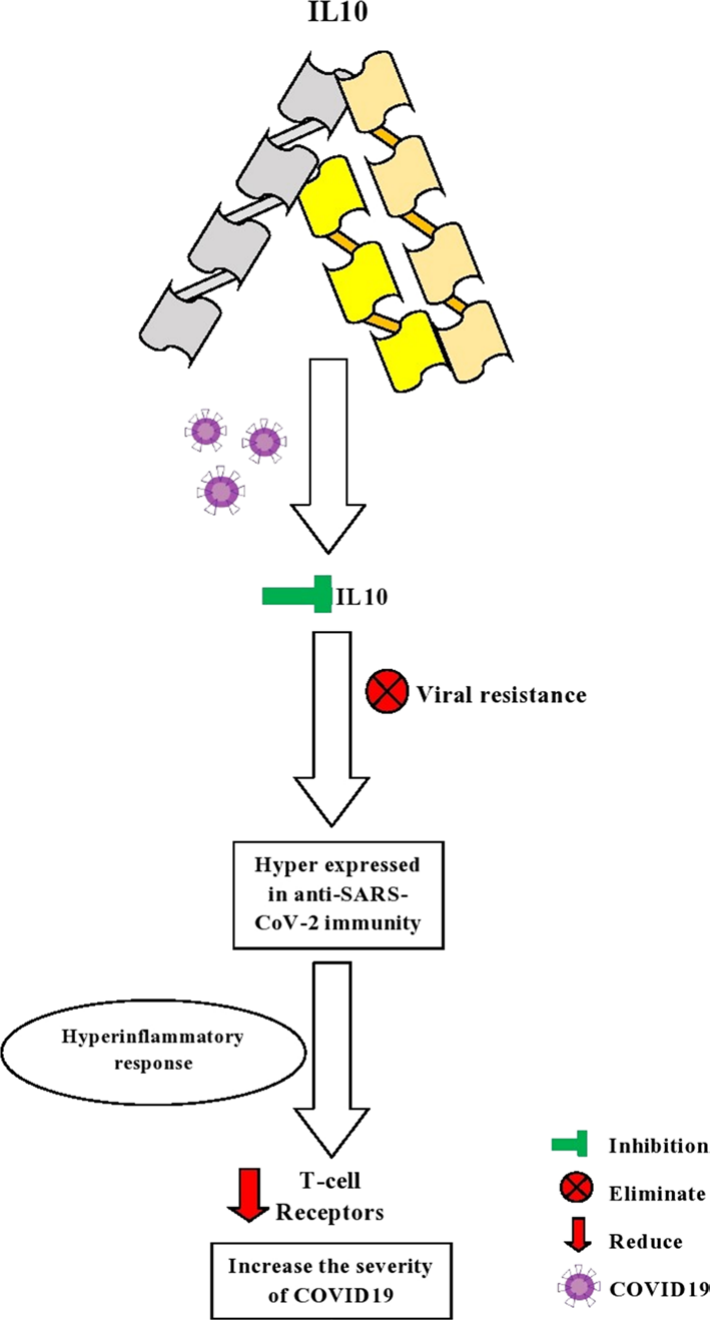
The severity of SARS-CoV-2 due to the interleukin-10 cytokine

**Fig. 10:**
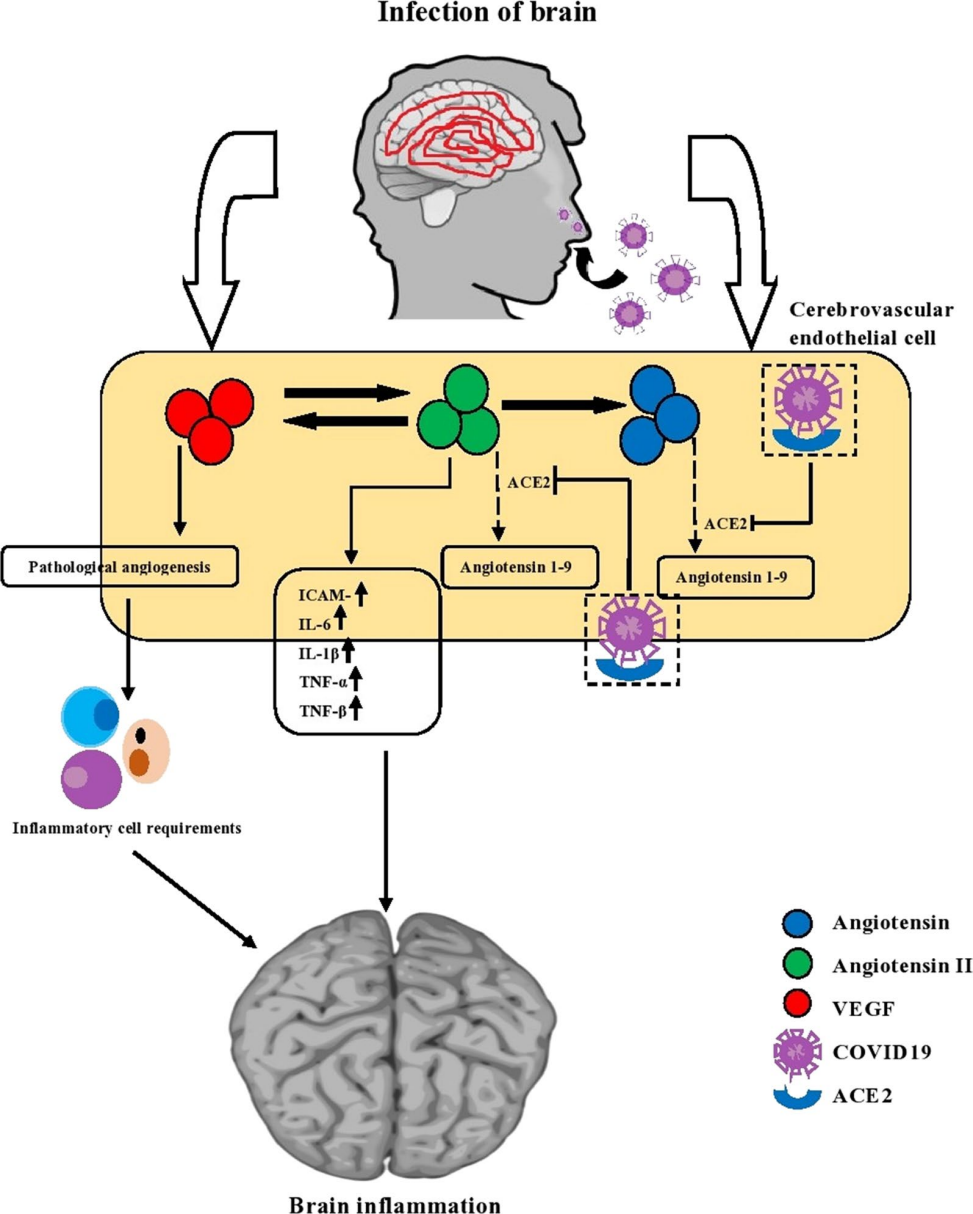
Mechanism of VEGF, ACE2 gene function in brain inflammation due to SARS-CoV-2

Figure 9 demonstrates that the SARS-CoV-2-infected brain (> 30 age) had decreased angiotensin 1–9/1–7 levels in cerebrovascular endothelial cells by depleting ACE2 in the improvement of angiotensin II type 1 receptor. It also the synthesis of VEGF, which pushes the inflammation in the central nervous system (CNS). In the meantime, the inflammatory cell employ becomes worsen by the VEGF synthesis, and pathological angiogenesis influences the proinflammatory, and there is a contrary level of angiotensin II increasing. This inflammation's side effects are nausea, anosmia, vomiting, headache, hemorrhagic stroke, disturbances of consciousness, altered mental state, acute necrotizing, seizure, etc. (Yin et al. 2020; Rodríguez-Puertas 2020) (Fig. 10).

## Impact of gene expression

Each selected genes have a differential expression on SARS-CoV-2 conditions and increase the disease severity. Expression in the ACE-2 gene affects the brain areas' function; IL-2, IL-7, IL-10 gene expression negatively regulates the transformation growth factor-β, alteration in TNF gene depletes the T lymphocytes, and VEGF gene expression causes the edema by the extravasation of immune cells (Table 3).

**Table 3:** Significant genes with their expression on SARS-CoV-2

S. no.	Major genes	Complications
1	ACE-2 (angiotensin-converting enzyme 2)	IT affects CNS and infects certain brain areas entering the olfactory bulb and thereby leading to loss of olfaction (Yin et al. 2020; Rodríguez-Puertas 2020)
2	IL-2, IL-7, and IL-10 (interleukins)	Autoimmune and immunostimulatory effects (Rodríguez-Puertas 2020). Negative regulation of transforming growth factor-β (Rousset et al. 2020)
3	TNF (tumor necrosis factor)	Depletion of T lymphocytes (Pellegrini et al. 2011)
4	VEGF (vascular endothelial growth factor)	Exacerbating edema and the outbreak of immune cells (Diao et al. 2019b)

SARS-CoV-2, which falls under the family of Coronaviridae, is highly contagious and has a higher rate of human–human transmission; the required method of transmission is through inhaling the droplets or coming in direct contact with virus surfaces. COVID-19 is a high risk to the population and healthcare workers worldwide. Many ideas for therapeutic options and vaccine development have been initiated, and also few countries have started to test the vaccines for SARS-CoV-2 infection. The four genes mentioned above contribute to the susceptibility of COVID-19, even though other factors influence, but genetic factors have a crucial role in disease severity. It is essential to emphasize the current issue with COVID-19 in which various genes show a positive correlation. As per the study conducted by a genome-wide association consisting of 1980 persons and they are infected with COVID-19 and other respiratory conditions, they have analyzed 8,582,968 SNP’s. The conclusion was made that gene cluster 3p21.31 is a genetic susceptibility in patients affected by the virus (Zhu et al. 2020). Hence, this assessment highlights the significant genes that focus on genetic changes and levels; ACE2 is a causative agent for coronavirus, and IL-2, 7, 10, TNF, and VEGF are involved in cytokine storm inflammatory response. These four genes are under study for coronavirus biology so that these genes may be used as potential biomarkers for early diagnosis and used for targeted drug delivery for COVID-19.

## Conclusion

This study encompassed the abnormal changes in the genes caused by the SARS-CoV-2 and pointed out the significant genes affected by this novel virus with the complete metabolic pathways. It is found that cytokines such as IL2, IL7, and IL10 play an essential role in maximizing the seriousness of the SARS-CoV-2. The ACE2 enzyme and VEGF affect the brain and cause inflammation in the central nervous system. The purpose of this review is to bring down the seriousness of the SARS-CoV-2. It is better to target the cytokines for the possible results, and for treating the SARS-CoV-2 neurological symptoms, VEGF is a probable therapeutic target in reducing inflammation in the brain. Therefore, this review could benefit not only for this virus's therapeutic basis but also for clinical observing, identification, and mediation of SARS-CoV-2 infection in the future.

## Data Availability

Not applicable.

## Abbreviations

SARS-CoV-2: Severe acute respiratory syndrome coronavirus 2
COVID-19: Coronavirus disease 19
WHO: World Health Organization
MERS: Middle east respiratory syndrome
ARDS: Acute respiratory distress syndrome
RNA: Ribonucleic acid
ASRD: Aspirin-sensitive respiratory disease
IL: Interleukin
TNF: Tumor necrosis factor
VEGF: Vascular endothelial growth factor
IHR: International Health Regulations
USA: United States of America
HAT: Human airway trypsin-like
NF: Nuclear factor
ACE: Angiotensin-converting enzyme
NK: Natural killer
WBC: White blood cells
TGF-β: Transforming growth factor-*β*
ICU: Intensive care unit
VEGFR: Vascular endothelial growth factor receptor
NS: Central nervous system
ALI: Acute lung injury
